# High-intensity focused ultrasound for biofilm debridement, an in vitro proof-of-concept using Ti-attached *Streptococcus mutans*

**DOI:** 10.1186/s40729-025-00645-3

**Published:** 2025-09-01

**Authors:** Minh Dien Tran, Sheetal Maria Rajan, Hien Chi Ngo, Amr Fawzy

**Affiliations:** Biomaterials Research Group, UWA Dental School, the University of Western Australia, Perth, Australia

**Keywords:** Titanium, Biofilm, *Streptococcus mutans*, HIFU, Ultrasound, Debride, Surface roughness, Peri-implantitis, Dental implant

## Abstract

**Introduction:**

Peri-implantitis (PI) is a biofilm-related condition driven by bacterial colonization on dental implant surfaces, leading to inflammation of the peri-implant connective tissue and progressive bone loss. Despite advancements, effective strategies for eradicating these biofilms remain elusive. While high-intensity focused ultrasound (HIFU) has been popularized in medicine, its effects on dental implant-attached biofilms remain unclear. This study presents in vitro findings on the effects of HIFU treatment on titanium (Ti)-attached *Streptococcus mutans* (*S. mutans*) biofilms and evaluates its impacts on the surface roughness and chemical composition of the Ti disc substrates.

**Methods:**

To optimise the HIFU parameters, four quadrants of a pair of Ti discs [machined (M) and alumina grit blasted (AB)] were marked using laser etching (MD Waterlase, US). HIFU beams, generated by a 254 kHz transducer and operated at intensities of 0 W, 10 W, 20 W, and 30 W, were applied to each quadrant for 2 min (min) in a water medium. The roughness of the treated surfaces was measured using Atomic Force Microscopy (AFM), and the surface composition was analyzed using Scanning Electron Microscope–Energy Dispersive Spectrometry (SEM–EDS). To investigate the biofilm debridement, 10-day-old *S. mutans* cultures were grown on 20 pairs of similar Ti discs, and then the optimized HIFU intensity of 20W was applied to five test pairs. Qualitative analyses were performed using a Dual Fluorescence/Reflection Confocal Laser Scanning Microscope (FRCLSM) and SEM imaging. Quantitative data on cell viability were collected using crystal violet (CV), (3-[4,5-dimethylthiazol-2-yl]-2,5 2,5-diphenyl tetrazolium bromide) (MTT), and flow cytometry (FCM) assays. Data from these test conditions were analyzed alongside cultures on biofilms that were untreated (control). Statistical data were calculated using ANOVA and appropriate *t*-tests for repeated measures.

**Results:**

The surface roughness of AB Ti discs showed a highest and significant increase (p < 0.05) following HIFU exposure at 20 W through three roughness parameters (Sa, Sq, and Sdr), compared to the controls (1207 nm, 1455 nm, 62% compared to 842 nm, 1042 nm and 30% respectively). This optimized HIFU treatment not only significantly reduced the bacterial counts of the biofilms (76% of total bacteria from M discs, 59% on AB discs in FCM assays) but also created areas of complete biofilm removal in SEM images.

**Conclusion:**

This study provides preliminary in vitro evidence that HIFU can remove bacterial biofilms. Further research is required to determine its feasibility as a potential non-surgical approach for the prevention and management of peri-implantitis.

## Introduction

Peri-implantitis (PI) can be defined as “a pathological condition occurring in tissues around dental implants, characterized by inflammation in the peri-implant connective tissue and progressive loss of supporting bone.” [1]. This distinctive pathology has emerged as a challenge in modern dentistry [2] due to the growing sheer number of dental implants placed annually [3, 4] combined with the high and progressive prevalence of the disease. In 2021, an estimated nine million dental implants were placed worldwide, amounting to a market value of $4.21 billion [5]. This value increased to $6.7 billion in 2024, with an expected annual growth rate of 8% from 2025 to 2030, according to Grand View Research [6].

Approximately one in five dental implants is affected by this pathology [7–10] to which current therapeutic approaches remain insufficient, with frequent recurrences that can ultimately result in the loss of the implant and supporting tissues [11, 12].

Although risk factors like smoking and diabetes contribute to the condition, complex bacterial biofilms have been identified as the primary pathogenic factor [2, 13–15]. Therefore, the primary therapeutic objective is to eliminate these bacterial aggregates. However, striving to achieve this goal is hindered by a major challenge—the intrinsic microbial resistance of multi-species oral biofilm communities [11, 16–18]. Consequently, mechanical and physical methods for biofilm removal have been extensively studied; however, these approaches remain constrained by notable limitations [19–23]. Recent reviews [12, 19, 20, 22, 24, 25] emphasize the necessity of mechanical decontamination for dental implant surfaces while underscoring the greater challenge of effectively debriding implants compared to natural tooth surfaces. Notably, the authors described how hand tools (titanium, plastic, Teflon, carbon-fiber) and mechanical instruments (abrasive air power systems, rubber cups, sonic tips) were effective as mainstream non-surgical approaches to reduce the clinical signs of PI, but these could not eliminate the disease. The use of air power abrasives (sodium bicarbonate, sodium hydrocarbonate, or the amino acid glycine), such as Airflow, has been shown in in vitro and in vivo studies to effectively remove the bacterial endotoxins and biofilms [26]. However, their application raises concerns, including aerosol generation, residual powder adherence to implant surfaces, and potential risks such as subcutaneous emphysema and epithelial desquamation, highlighting the need for cautious implementation and further investigation. Similarly, metal ultrasonic scalers equipped with metal tips have been shown to reduce dental plaque accumulation and create a smoother implant surface. However, the extent of plaque reduction remains modest (73–53%), and concerns persist regarding their potential hyperthermia effects on dental implants [26]. Additionally, in vitro studies have explored the application of electric currents, with a recent review by Rodrigues et al. [27] showing promising results.

In addition to these treatments for managing PI, other approaches—such as the topical application of chlorhexidine, lasers, photodynamic therapy, and systemic probiotics—have been explored as adjunct therapies and require further investigation [12, 24, 26]. Nevertheless, the plethora of current treatment methods for PI does suggest that a variety of clinical management strategies are required to manage the condition from patient to patient. Towards this goal, one novel approach to debridement of dental implants to manage PI is the use of high-intensity focused ultrasound (HIFU). This technology offers benefits over existing methods in that it is devoid of radiation, free from aerosols, requires no additional materials, and can effectively reach inaccessible irregularities of the target.

Since the first article was published in 1927 by Wood and Loomis [28], followed by works of Lyn et al. [29] and Fry brothers [30] HIFU has been successfully implemented to treat a variety of medical conditions including prostate cancer, Parkinson’s disease as well as neuropathic pain [31] through exploitation its mechanical ablation and thermal coagulation properties [32, 33]. In medical therapies, HIFU is a non-invasive technique that concentrates ultrasound energy on a small target to induce hyperthermia or ablation via cavitation [34, 35]. Therapeutic frequencies range from 300 kHz to 7 MHz, with intensities typically exceeding 1500 W/cm^2^—far greater than diagnostic ultrasound—and exposure times ranging from seconds for focal ablation to 60–90 min for multi-site treatments. Clinically, HIFU is applied under ultrasound or MRI guidance for tumour ablation (e.g., prostate, liver, uterine fibroids), neurosurgical tremor control, targeted drug delivery, and aesthetic interventions such as skin tightening [34, 35]. The balance between frequency and intensity determines whether hyperthermia or ablation predominates, meaning lower frequencies (500 kHz) enable deeper penetration and greater ablation with reduced near-field heating, whereas higher frequencies produce more superficial thermal effects with limited mechanical ablation [34–37].

Despite the popularity in medicine, the use of HIFU in dental research and clinical therapies is underdeveloped [38]. Nevertheless, the level of interest in this domain has seen a two-fold increase in research volume on this subject between 2010 and 2020, in contrast to the preceding decade (as indicated by a Google Scholar search utilizing the keywords “dentistry AND high intensity focused ultrasound”) [38]. Amongst other findings [39–43], microbiology–related studies have shown that HIFU could inhibit *S. mutans* growth on dentine [42, 44] and eliminate or destroy *Escherichia coli* (*E. coli)* and *Enterococcus faecalis* (*E. faecalis)* biofilms [43, 45]. A recent study by Rajan et al. [46] demonstrated HIFU’s ability to inhibit the metabolic activity of *E. faecalis* biofilm. Despite these advancements, the potential application of HIFU for detaching bacteria from dental implants remains inadequately characterized. Here, we investigate the effects of HIFU on the removal of *S. mutans* biofilms attached to Ti substrates, as well as the impact of HIFU on Ti substrate surface topography. We hypothesised that HIFU could change the Ti surface roughness and effectively remove the biofilms. The accepted hypothesis could have implications for our understanding of the application of HIFU for Ti dental implants and the clinical management of PI.

## Materials and methods

### Study design

#### Ti substrates

A total of 22 pairs of Ti discs (10 mm in diameter, 78.54 mm^2^ surface area, and 2 mm thick) and a pair of Ti implants (4 mm in diameter, 9 mm in length) [DCT4009 DC Unroughened (machined—M) and DCT4009 DC Roughened—R)], manufactured by Southern Implants (Pty Ltd., Irene, South Africa), were utilized in this study. The biofilm growth side of the discs was machined (M) on 22 discs and roughened using alumina grit blasting (AB) on the other 22 discs. The remaining surfaces of the discs were polished to minimize bacterial adhesion.

**Fig. 1:**
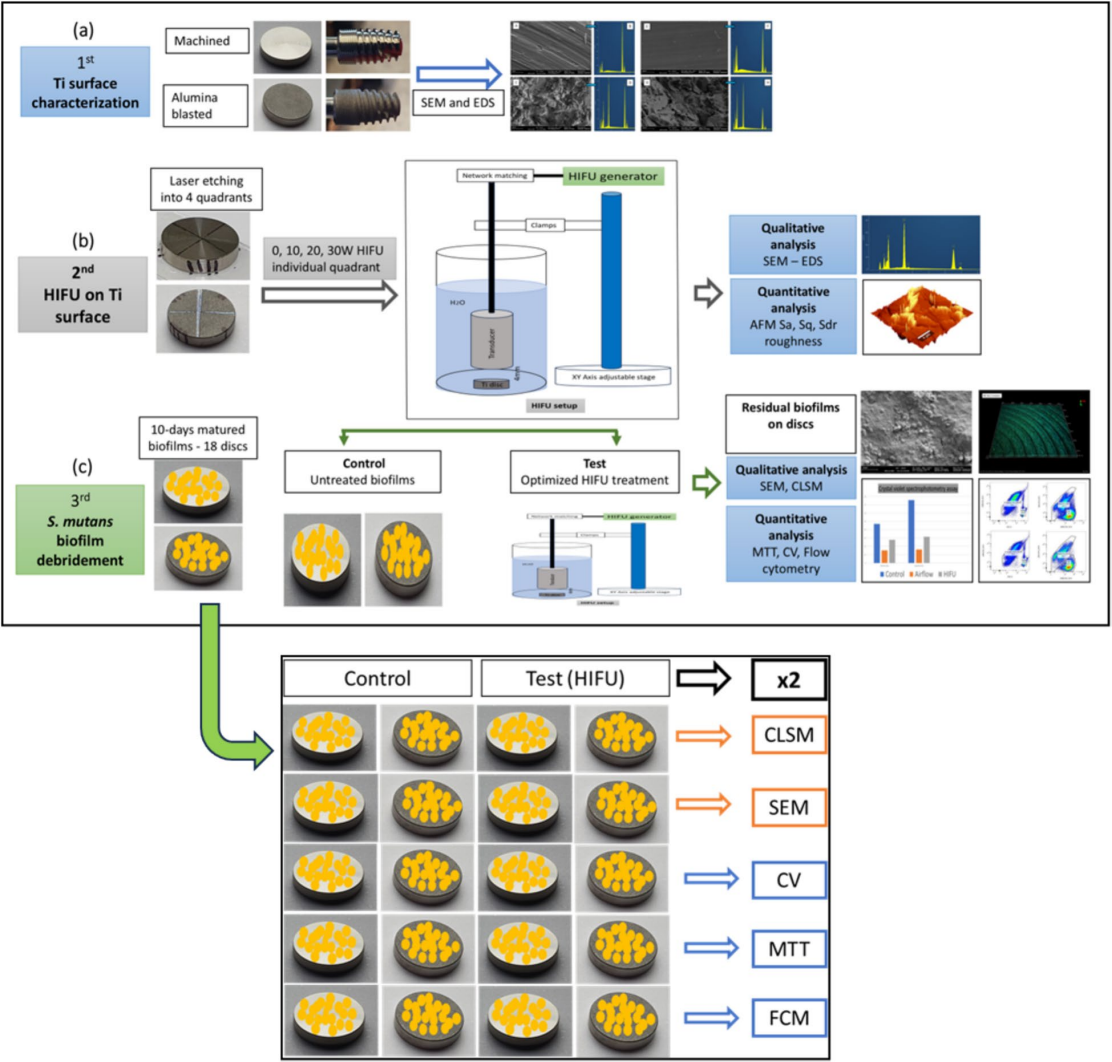
**Above**: the study design includes: **a** characterization of the Ti test surfaces through qualitative analysis using SEM and EDS, along with quantitative surface roughness assessment via AFM; **b** evaluation of the effects of different HIFU intensities on substrate surface roughness and chemical composition; and **c** investigation of the debridement effects of HIFU on *S. mutans* biofilms grown on Ti substrates. **Below**: number and grouping of the samples in the HIFU biofilm debridement stage

Two sets of discs and one set of implants were utilized for surface characterization and assessing HIFU effects on microscopic topography and chemical composition, while the remaining 20 sets were used for HIFU biofilm debridement investigation. The methodology is illustrated in Fig. 1.

#### Study design

The study workflow is visually summarized in Fig. 1, depicting three interconnected investigative stages. The first stage involved the surface characterization of Ti discs using three atomic force microscopy (AFM) roughness parameters: Sa (arithmetic mean height), Sq (root mean square height), and Sdr (developed interfacial area ratio). Additionally, scanning electron microscopy (SEM) and Energy Dispersive X-Ray Spectroscopy (EDS) were employed to analyze the microscopic topography and chemical composition, with results compared to those of commercial Ti implants from the same manufacturer.

The second stage focused on optimizing HIFU by assessing the effects of varying HIFU intensities on Ti surface roughness and chemistry. Lastly, the third stage examined the debridement of *S. mutans* biofilms attached to the Ti surface.

For biofilm characterization, two qualitative imaging techniques—dual fluorescence/reflection confocal laser scanning microscopy (FR-CLSM) and SEM—were used to visualize the biofilm topography on Ti surfaces. Additionally, three quantitative methods—crystal violet (CV), MTT, and flow cytometry (FCM)—were employed to assess different biofilm properties. Specifically, the CV assay quantified biomass, the MTT assay measured metabolic activity, and FCM determined the number of bacteria remaining on Ti surfaces after treatment. The results were compared to control samples, where biofilms were left untreated.

### HIFU setup

A HIFU transducer (XDR094 S/N:19, Sonic Concept, USA) of 15.5 mm in diameter was connected to a TPO-200-09 HIFU generator (Sonic Concept, USA) through a XDR094-019 matching network. The mechanical index of the HIFU output was calculated to ensure it remained within the therapeutic range using the equation $MI \, = \, P_{ra} /\sqrt {fc}$. The transducer was operated at a frequency of 254 kHz, pulse mode (3 ms burst length/6 ms cycle), duration of 2 min, and with varying output power of 10W, 20W, and 30W, respectively. The test Ti surfaces were positioned in a 25 mL beaker filled with distilled water, and the transducer was positioned at 4 mm (focal length)*.* Temperature change occurring during HIFU application on the Ti surface was measured using a thermocouple (Lutron BMT-4208SD, Taiwan).

### Ti surface characterization with and without HIFU exposure

The Ti surfaces were marked into 4 quadrants using laser etching (Er, Cr: YSGG MD biolase, Cromwell Irvine, CA, US). Only quadrants 2, 3, and 4 were subjected to HIFU parameters while quadrant 1 was used as a control. The unexposed quadrants were covered with Polyvinyl Siloxane impression material (3 M ESPE Imprint^™^ light) to avoid overlap of HIFU exposure.

The microscopic roughness, topography, and surface chemistry of each quadrant were characterized following the protocols detailed in Sect. "Study design".

The surface roughness and topography of the Ti disc *(n* = *1 pair*) were analysed using AFM (WITec alpha 300RA +—Germany). AFM was performed at high resolution in tapping mode using a silicon cantilever coated with aluminium. An area of 20 µm × 20 µm [40] was scanned with a resolution of 512 lines and a speed of 2 s. Data was collected and analysed using Project5 5.2 (WITec Suite *FIVE* software—Germany). The background–subtraction Sa, Sq, and Sdr nanoscopic roughness data from discrepancy–free scans were extracted from topography mode for data analysis [47].

SEM–EDS  was utilized to examine the microscopic topography and chemical composition. The selected Ti disc pair and a pair of Ti dental implants were mounted on aluminum stubs using Cu tape and coated with a ~ 20 nm carbon layer (Polaron SC7640, Quorum Technologies Ltd, UK).

For each sample, 10 random sites were selected for analysis. SEM images were captured at magnifications of 500 × and 5000 ×, followed by EDS data acquisition at 20 kV with a 60 µm aperture using a secondary electron detector. The EDS data was then analyzed using AZtec^®^ 5.1 software (OXFORD Instruments, UK).

### HIFU biofilm debridement effects

#### Biofilm formation

This protocol was modified from Lemos et al. [48] and ATCC bacteriology culture guide.

*S. mutans* (ATCC 700610) were grown overnight in Brain–Heart Infusion broth (BHI) (Sigma-Aldrich, Australia) supplemented with 1% sucrose (Sigma-Aldrich, Australia) at 37 °C for 24 h and adjusted to a concentration of 5 × 10^6^ colony-forming units (CFU) per mL (optical density at 600 nm = 0.5) (McFarland Densitometer–Fisher Biotec Australia).

10 pairs of M and R Ti disc were placed individually in a 24 well plate (× 2) with 2 mL of adjusted bacterial suspension and incubated at 37 °C for 10 days in an orbital shaker at 50 rpm, the medium was replenished every 2 days. On day 10, Ti discs were washed gently in 2 mL of phosphate-buffered saline (PBS) to remove loose bacteria and placed in 2 mL of PBS until treatment.

#### Biofilm debridement protocol

Based on the results obtained from the second stage (2.2. section), the HIFU parameters were optimised to be further operated at the following conditions: 254 kHz frequency, pulsed mode (3 ms/6 ms), 2 min at 20 W. The discs were divided randomly into four experimental groups: Control (M surface), Control (AB surface), Test (M surface), and Test (AB surface) with five discs each (*n* = 5 each) (Fig. 1).

For HIFU treatment (*n* = 5 × 2), the discs containing biofilms from the test groups were placed in 25 mL glass beakers filled with 10 mL of PBS. The HIFU transducer was hovered over the biofilm surface at 4 mm and operated at the above-mentioned parameters. After each application, the transducer was kept in 70% Ethanol for 10 min for disinfection followed by dipping twice in PBS for 2 min each to remove the alcohol.

The control samples (*n* = 5 × 2) were not exposed to HIFU.

The treated discs were gently dipped 4 times in 2 mL of PBS to remove debris and placed in 2 mL of fresh PBS. The number of each experimental group was selected randomly into two analysing subgroups for qualitative (*n* = 2 pairs) and quantitative *(n* = 3 pairs) analyses. The experiment was then repeated once, resulting in a total sample of 20 pairs. The number and grouping of the sample are summarized in Fig. 1.

#### Biofilm characterization protocol

##### Qualitative characterization

###### Confocal laser scanning microscopy (CLSM)

Four Ti discs, one from each of the four experimental groups (*n* = 2 pairs of M and AB), were stained with LIVE/DEAD^™^ BacLight^™^ following the manufacturer’s instructions. The images of the stained biofilms were visualised using CLSM. A dual fluorescent/reflection CLSM Z stacks from random sites of each disc were taken at 10x (NA 0.45, 1024-pixel size) with the laser wavelength 488/561 nm for fluorescent and 405 nm wavelength for reflection using Nikon A1 Si Confocal Microscopy (Nikon Instruments Inc, NY, USA). The reflected light was collected between 405 and 750 nm wavelength range.

###### Scanning electron microscopy (SEM)

After fixing the biofilms with 4% paraformaldehyde, four Ti discs, one from each of the four experimental groups (*n* = 2 pairs of M and AB), were affixed onto aluminium stubs using Cu tape and carbon coated (~ 20 nm) (Polaron SC7640, Quorum Technologies Ltd, UK). A total of 10 sites were randomly selected for examination from each disc, and the SEM images were captured at a magnification of 5000 × at 5 kV, 30 µm aperture using a secondary electron detector.

##### Quantitative characterization

To release the biofilms from the attached Ti surfaces, the samples (*n* = 6 pairs) were each placed in 5 mL flat-bottom tubes filled with 2 mL of PBS and placed in an ultrasonic bath (L&R SweepZone Technology, NJ, USA) for 10 min. The tubes were then shaken using a vortex mixer for 2 s, the 2 mL suspension was extracted for CV, MTT, and FCM assays.

###### Crystal violet assay (CV)

The Ti discs from each group (*n* = *2 pairs*), after treatment, were assessed using the CV assay, following a method previously described with minor adjustments [49]. 100 µL of suspension was pipetted onto 96 well plates, and 100 µL of 0.1% aqueous crystal violet solution (CV; Sigma-Aldrich, Australia) was added into each well and incubated for 15 min. Afterward, the CV solution was gently aspirated, and the specimens were washed three times with distilled water. The biofilm was then solubilised with 30% acetic acid, and the CV was quantified by measuring the absorbance at 590 nm using a microtiter plate reader (Sunrise ^™^, Tecan, Switzerland).

###### MTT assay

To further investigate the metabolic activity of the viable bacteria, (3-[4,5-dimethylthiazol-2-yl]-2,5 diphenyl tetrazolium bromide) MTT assay was performed using bromide kit (0.5 mg/mL MTT solution) (Sigma-Aldrich, Australia), in accordance with the manufacturer’s instructions. 100 µL of the bacterial suspension obtained after ultrasonication was then pipetted in triplicates into a 96-well plate in a volume of 100 µL. Then, 10 µL of MTT reagent were added to each well, and the plate was covered and incubated at 37 °C for 4 h. Following incubation, the reagent was aspirated, and 100 µL of the solubilizing solution was added, and subjected to incubation overnight at 37 °C. The absorbance at 600 nm was measured using a spectrophotometer (SunriseTM, Tecan, Switzerland).

###### Flow cytometry analysis (FCM)

For bacterial counts using FCM**,** 1 mL suspension was centrifuged at 4000 rpm for 10 min, the supernatant was removed, and 2 mL of 0.9% sodium chloride was added to the tube. After vortex mixing, 100 µL of this bacterial suspension was extracted for staining with SYTO9 and propidium iodide from LIVE/DEAD^™^ BacLight^™^ Bacterial Viability and Counting Kit (Thermo Fisher Scientific) following manufacturer’s instructions (100 µL bacterial suspension + 1.5 µL propidium iodide + 1.5 µL SYTO9 + 10 µL microspheres + 887 µL sodium chloride 9%) producing 1000 µL of stained bacterial suspension for measurement [47].

Four single-color controls were prepared, and the results from these control samples were used for voltage calibration and gating. An LSR Fortessa cytometer and BD FACSDiva^™^ software (BD Biosciences) were used with laser wavelengths of 488 nm and 561 nm. The fluorescence signals were collected in three channels with the filters of 610 nm (for 561 nm laser), 530 nm, and 695 nm (for 488 nm laser). Forward scatter, side scatter, and fluorescence data were collected for 5 min using a medium flow with logarithmic signal amplification, and the files were saved in FCS (flow cytometry standard) format and analysed using FlowJo v10 (BD Biosciences) software. The sample quality was controlled using the inspect function of the software, the FSC versus time plot, and the number of beads (10^6^ in each 1 mL sample). The four single-color controls were used for gating, which differentiated three populations of SYTO9 stained (live), PI stained (dead), and total bacteria. The total bacterial counts and the percentage of live versus dead bacteria were collected as data for statistical analysis [47].

### Statistical analysis

Data were presented as mean ± standard deviation and analysed using ANOVA followed by *T* tests (Microsoft Excel and R statistical software). Statistical significance was measured at p < 0.05.

## Results

### Ti surface characterisation without HIFU exposure

**Fig. 2:**
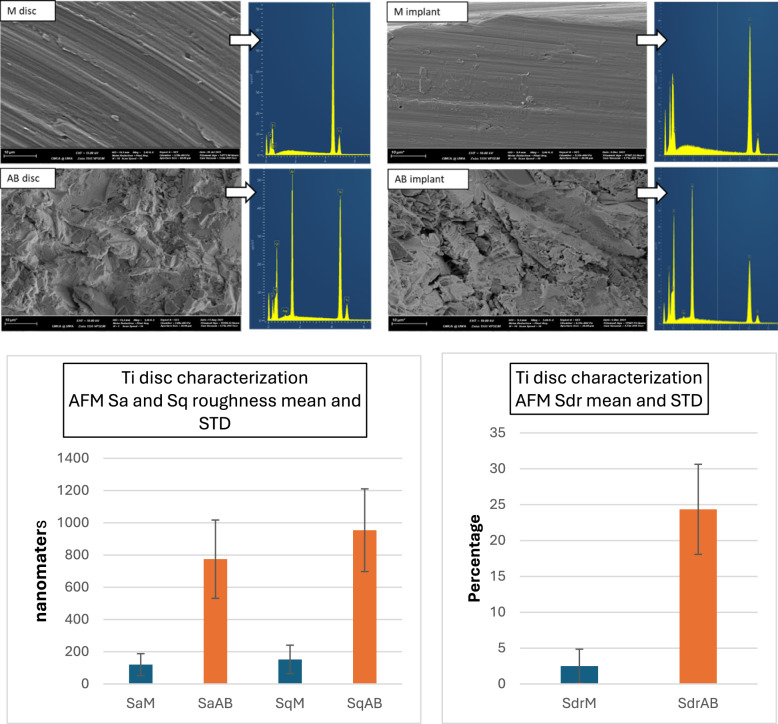
(Above) Qualitative data SEM images of M disc showing concentric lines and EDS with predictable Ti, O, and C present. SEM image of AB discs displaying a high level of roughness and irregularity with the addition of Al in EDS. (Below) Quantitative data AFM surface roughness supporting the SEM with AB disc showed much higher Sa, Sq, and Sdr. Compared to the surface of the dental implants from the same manufacturer (E, F, G, H)

Results from SEM and EDS analyses were presented in Fig. 2. M discs were characterised by concentric cutting lines of 1 µ apart with a noticeable level of heterogeneity in depth, width, and continuity. Debris, scratch lines, and voids were also observed. Those of AB discs showed patterns of high-level irregularity with sharp troughs and peaks which appeared less than 10 µ in height. Debris was less distinguishable on the rough background. Aluminium was detected on AB Ti surfaces apart from the expected carbon (C), Ti, and oxygen (O). When compared to the SEM images of Ti dental implants of the same type and manufacturer, EDS showed an identical pattern while the machined surface of the implant appeared significantly smoother than the M disc.

### Ti surface characterisation with HIFU exposure

#### SEM and EDS

**Fig. 3:**
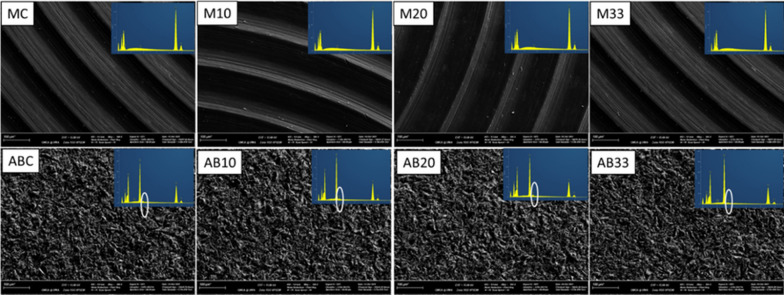
SEM images and EDS analysis of the 4 quadrants from M and AB Ti discs above. There were no noticeable differences in SEM images. Traces of Si were detected only on AB surfaces

Results from SEM and EDS analyses were presented in Fig. 3. SEM images showed no noticeable differences amongst the four quadrants of the same Ti disc. However, silicon (Si) was detected on the AB surfaces in addition to O, Ti, aluminium (Al) found in the characterization step. The presence of Si was of low value but consistent across EDS series.

#### AFM surface roughness

**Fig. 4:**
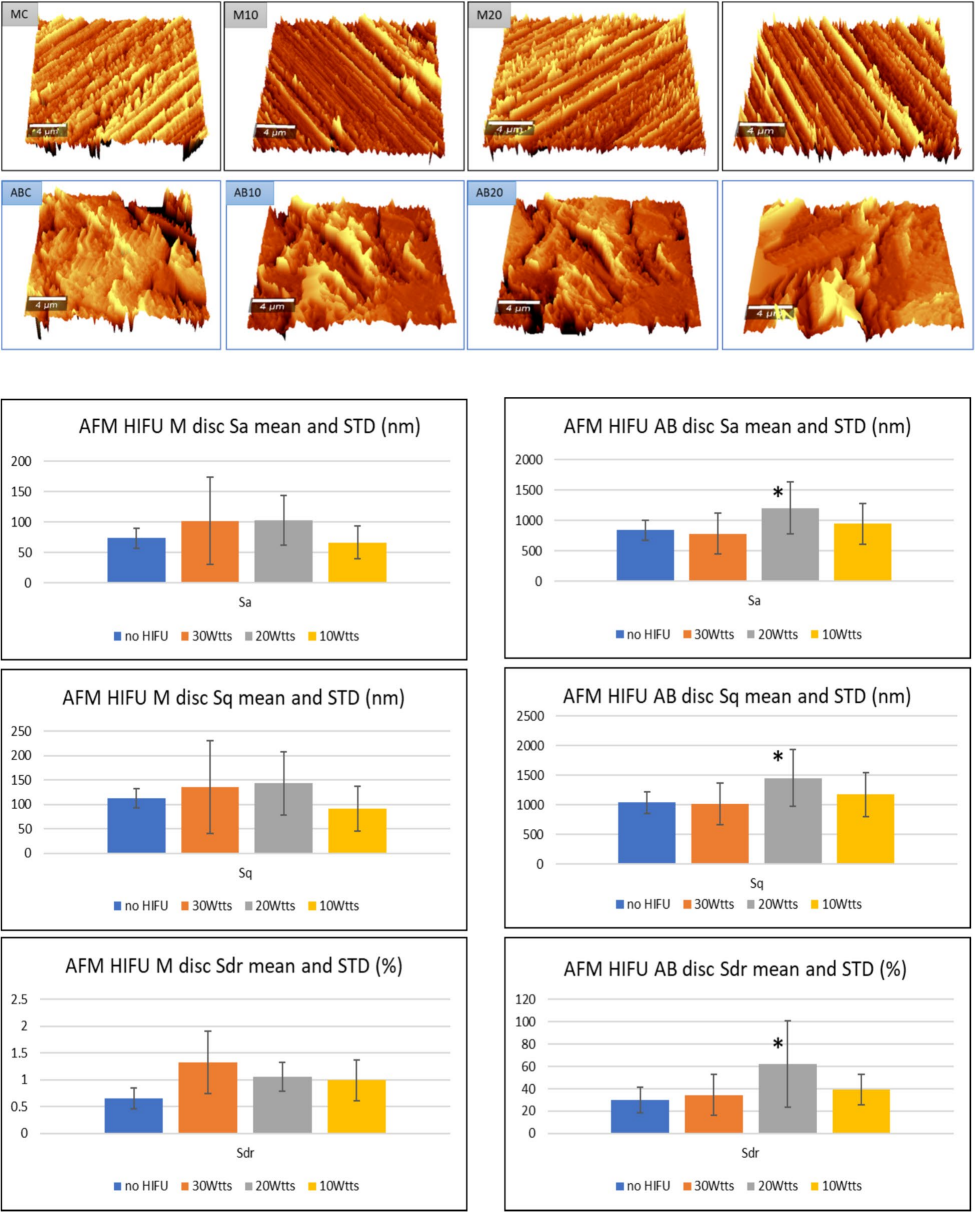
3D images representing the amplitude of the AFM scan in tapping mode (above) and the roughness parameters collected from the four quadrants of the two discs. Significant differences (*) in all three surface roughness parameters (Sa, Sq and Sdr) were observed in the 20W HIFU treated AB surface (below)

Results from AFM analysis were presented in Fig. 4. AFM Quantitative analysis of the Ti surfaces, AB disc demonstrated consistently much higher (≥ four times) values in all three roughness parameters of Sa, Sq, and Sdr compared to M disc. HIFU treatment did not change these three parameters except in the 20W group on AB surfaces compared to the control, and the differences were statistically significant.

### HIFU biofilm debridement effects

#### Qualitative SEM and FRCLSM images

**Fig. 5:**
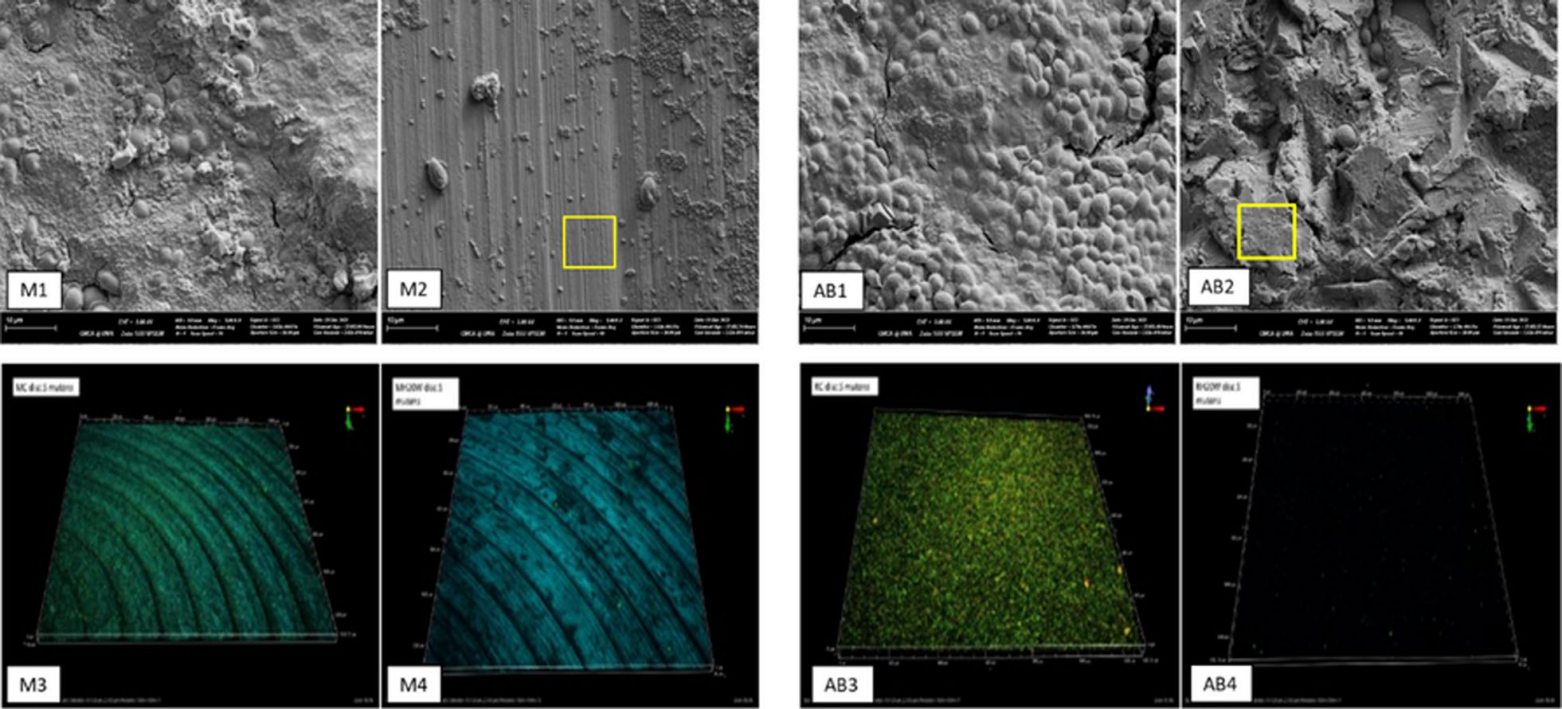
reflection SEM (M1, M2, AB1, AB2) and 3D projections of the dual fluorescent CLSM images (M3, M4, AB3, AB4) of the *S. mutans* biofilms of control and test groups. In addition to the significant breakdown and reduction of biofilms, test surface also showed areas of total devoid of biofilms (yellow squares)

Results from SEM and FRCLSM analyses were presented in Fig. 5. Dual fluorescent–reflection CLSM 3D projections of the Z stacks showed thicker *S. mutans* biofilms on the AB surfaces compared to the M substrate. HIFU appeared to have the ability to remove biofilms from both discs. Even though the removal was incomplete on SEM images, there were areas totally devoid of the biofilms.

#### Quantitative MTT, CV and FCM assays

##### MTT and CV assays

**Fig. 6:**
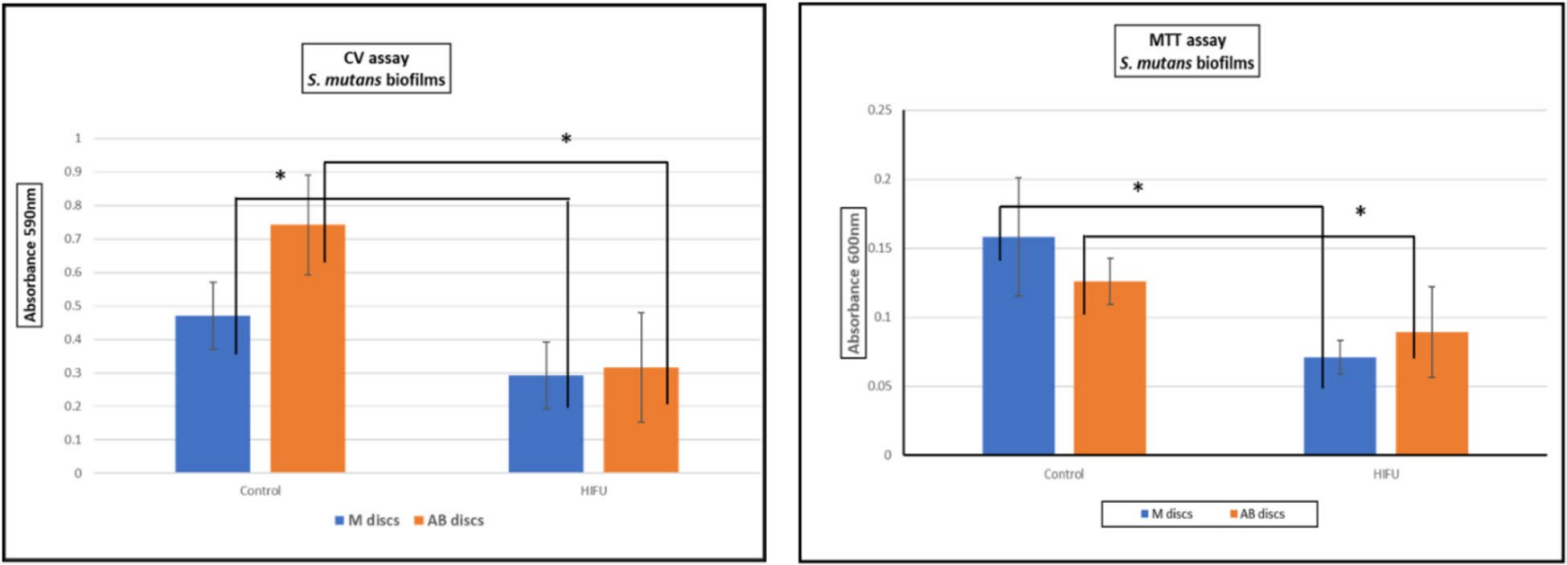
CV and MTT assays showing the statistically significancant (_*_) reduction in both biomass and metabilic activity in the HIFU-treated (test) group compared to the control

Results from MTT and CV assays were presented in Fig. 6. These two assays exhibited a consistent pattern of the reduction of the absorbance values in the test groups with statistical significance. The AB surfaces appeared best to facilitate the growth of the biofilms with the highest CV assay value which represents the total biomass. However, the live bacteria appeared to thrive most on M surface with the highest MTT assay value which is designed to measure the metabolic activity of the biofilms. Interestingly, there was no significant difference in both MTT and CV measurements of residual biofilms between M and AB surfaces on the contrary to SEM images.

##### FCM assays (Fig. 7)

**Fig. 7:**
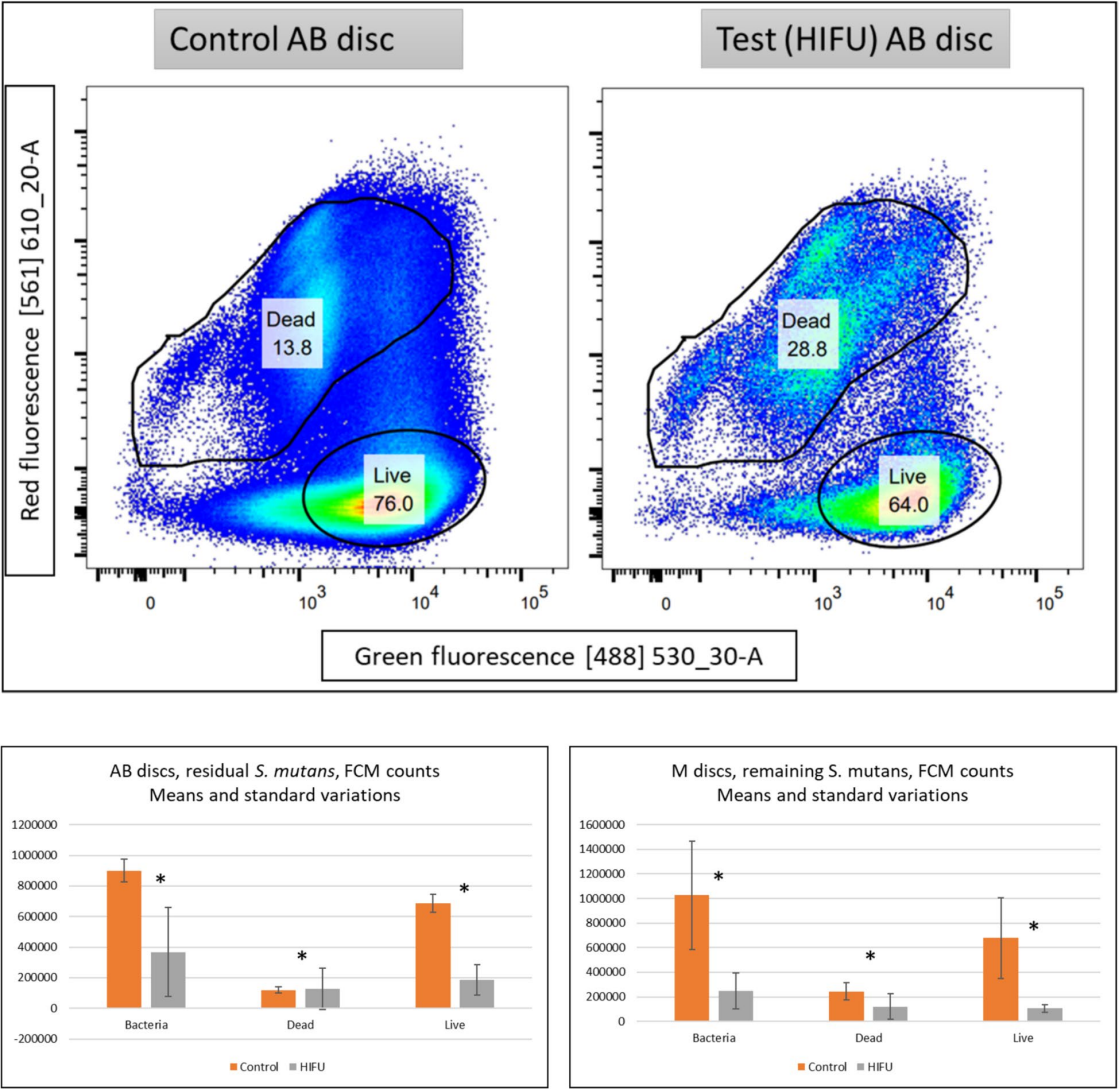
(Above) Pseudo colour flow cytometry plots displaying the two populations of “live” and “dead” bacteria stained with LIVE/DEAD^™^ BacLight^™^ Bacterial Viability and Counting Kit. Live bacteria absorbed SYTO9 and emitted green fluorescence from 488 nm wavelength laser excitation, while PI-stained dead ones emitted red fluorescence from 561 nm channel. (Below) the means and standard variations of the bacterial counts showing the similar statistical difference (_*_) appeared in MTT and CV assays

Results from FCM assay were presented in Fig. 7. Regarding FCM flow, 450 µL of the suspension containing 180,000 microsphere on average passed through the cytometer in the 5 min of data collection.

In FCM, the control biofilms had the higher counts in which predominantly SYTO9 stained (live) bacteria. Lower total counts were observed in the HIFU-treated test samples. The difference between the two experimental groups were statistically significant in the similar pattern seen in MTT and CV assays, proving the debridement effects. The SYTO9 green fluorescence expression was high and much more focused in a shorter range than PI, which spread out along both two axes. These two bacterial populations were more distinguishable in the test group compared to the control samples, which showed a bridging region with modest fluorescence emission and density.

## Discussion

The main goal of this study was to examine the debridement effect of HIFU on *S. mutans* biofilms attached to flat titanium surfaces that mimic the microscopic topography of Ti dental implants. Our findings, obtained through various measurement techniques, accept the hypothesis that HIFU, when applied at an optimized intensity, can effectively remove biofilms attached on both machined and sandblasted Ti surfaces similar to commercial implants. Additionally, we found that HIFU at a specific intensity (20W) can enhance the roughness of the alumina grit blasted (roughened) Ti surface.

Our main finding can be explained by the ablation property of ultrasound above the diagnostic mechanical index of 1.9 [50]. To calculate the mechanical index in our HIFU setting, we use the equation $MI \, = \, P_{ra} /\sqrt {fc}$ [50] where MI is the mechanical index; *P*_*ra*_ is the peak negative pressure in MPa which is in excess of 2 MPa according to the HIFU generator manufacturer; and *fc* is the central frequency of 0.254 MHz. our MI value is ≥ 3.968. The hyperthermic effect of HIFU is exceedingly unlikely the cause for the finding as the highest temperature measured from Ti surfaces did not increase more than 4 °C during HIFU application.

Despite the lack of direct evidence associating *S. mutans* with PI [13], this bacterial species is indigenous to the oral cavity and abundant in both supra- and subgingival sites [51, 52], and plays a key role in oral biofilm establishment. As of 1st November 2024, *S. mutans* accounted for 11,488 PubMed-indexed publications (0.78% of all bacteria-related literature), ranking as the 15th most studied bacterium [53]. First identified in 1924 by J. Kilian Clarke [54], *S. mutans* is a Gram-positive, facultative anaerobe within the mutans streptococci group, well established as a principal pathogen in dental caries, and a key contributor to the initiation and progression of oral biofilms [52, 55–60]. This species grows rapidly, with an exponential rate of 0.24 h^−1^ [61], and exhibits distinctive pathogenicity that supports both its own colonization and the development of complex biofilm-associated microbial communities [52]. Its biofilm-forming capacity relies on extracellular polysaccharide (glucan) production via glucosyltransferases, glucan-binding proteins, collagen-binding proteins, and sucrose-dependent adhesins, alongside quorum-sensing systems [52, 59]. According to Fürst et al. (2007), bacteria colonized implant surface immediately after the placement [62], and the early colonization involved Gram-positive aerobes or facultative anaerobes [57], with *S. mutans* demonstrated strong ability to initiate biofilm formation during the first 16 h and extend into the early colonization through salivary pellicle binding [58, 60]. This pioneer colonization facilitated subsequent integration of anaerobic PI-associated pathogens, such as *P. gingivalis* and *F. nucleatum*. As biofilms mature, streptococci reemerged as dominant species, comprising up to 25% of bacterial counts [55, 60]. Given its central role in biofilm dynamics and ability to form highly reproducible biofilms, *S. mutans* was selected as the test organism for this mono-species, proof-of-concept in vitro study.

The imperfect debridement capability of HIFU in this study can be explained by the lack of visual guidance and the lower negative pressure (≥ 2 MPa) compared to other studies using 4.1–7.6 MPa values [45]. Without a guidance system, we selected to place the transducer and Ti disc surfaces parallel to each other with the view that the ultrasound beams would strike the targets at the right angle, which may not be the optimized direction. We believe that biofilm debridement effects could be improved with more focused, powerful, and beam-guided transducers. A handpiece-like HIFU delivery system, featuring a HIFU transducer using running water as both a transmission medium and coolant, has been conceptualized as a potential clinical tool and is proposed for development in the next phase of research.

Although rarely used for visualising biofilms attached to dental implants since it was reported by Baschong et al. in 2001 [63], the dual fluorescent and reflection CLSM technique proved to be particularly valuable in this study. This method not only allowed the visualization of adhered biofilms but also revealed their distribution concerning the Ti surface topography. Another key advantage of this technique was its ability to focus laser beams on depleted biofilms. Since most biofilms were removed by HIFU, the remained bacteria emitted insufficient fluorescence for detection, making it extremely challenging to locate the samples. However, using the reflection CLSM mode, this process became significantly easier and more precise.

Through a combination of SEM, EDS and AFM analysis, the roughness parameters of Sa, Sq and Sdr of the roughened surface were significantly increased (p ≤ 0.05) at the specific HIFU intensity of 20W in 2 min. Concerning the Ti surface roughness, the greater effect observed at 20 W, compared with 10 W and 30 W (Fig. 4), merits further investigation. In principle, the ablation effect of HIFU increases with both intensity and exposure duration, indicating that these parameters may influence surface roughness. In light of the scarce evidence regarding HIFU’s influence on this property of Ti, we interpret the 20 W, 2 min treatment as likely dislodging surface impurities and thereby exposing finer microscopic irregularities. At higher intensities, further surface alterations may occur. In the absence of quantitative analysis, we can only hypothesize that the observed increase in roughness was driven by the cavitation and ablation effects of HIFU, which, at this specific setting, removed contaminants originating from the alumina grit-blasting process used to roughen Ti surfaces during manufacturing. These findings, however, contrast with those of Sedlaczek et al. (2017), who reported that low-frequency ultrasound applied for a substantially longer duration (25 kHz for 10 min/cm^2^) reduced the Sa surface roughness of sandblasted–acid-etched (SLA) Ti surfaces. This reduction was attributed to the removal of surface carbon contamination caused by the ablation effects of ultrasound [64].

Due to the limited research on HIFU’s effects on Ti surface roughness and the conflicting findings between the two studies, further large-scale investigations with different HIFU settings, parameters and Ti surface topographies are needed to confirm its impact on Ti surface roughness and composition.

Regarding biofilm debridement, there is a lack of similar studies using HIFU in the literature. Therefore, we compared our findings with studies that examined the effects of ultrasound cavitation. Two *in-vitro* experiments conducted by Vyas et al. [65–67] using high-speed camera observations illustrated that cavitations produced by ultrasonic scaler tips oscillating between 25 and 50 kHz could effectively disrupting and eliminating *Streptococcus sanguinis* biofilms adhered to glass coverslips, titanium discs, and implant surfaces. Study by Yamada et al. [68] utilizing a comparable imaging qualification method showed cavitation water jets were capable of removing plaque biofilms developed on rough dental implants worn in the mouth of four volunteers for a duration of 72 h. These investigations support our understanding that HIFU cavitations have positive biofilm debridement effects.

Given the strong evidence for the aetiological role of microbial biofilms in peri-implant diseases, thorough biofilm removal is essential for effective disease control. Mechanical and physical approaches—including mechanical instrumentation (titanium curettes, air-abrasive devices), laser-assisted therapy (e.g., Er:YAG, diode), and photodynamic therapy—have been widely investigated [19–23], yet each presents notable limitations. Recent reviews have underscored the difficulty of debriding implants compared with natural tooth surfaces [20, 69–72], attributable to the implants’ complex three-dimensional architecture, prosthetic components, and, most critically, peri-implant pockets [73]. Three systematic reviews of clinical studies published in 2023 [20, 70, 71] reinforced these findings. These conventional methods deliver energy in a pulsatile or clustered manner and require unobstructed access, meaning localized, intermittent application may leave areas untreated.

In medical contexts, HIFU can penetrate solid barriers, such as human tissues, and accurately focus on deep targets to produce ablation effects [35]. Unlike existing mechanical and physical debridement methods in dental implantology, HIFU delivers acoustic energy that propagates through a three-dimensional transmission medium. Given that peri-implant pockets are fluid-filled, low-frequency HIFU could, in principle, penetrate peri-implant soft tissues and interact with the complex three-dimensional topography of titanium implant surfaces. However, this potential remains hypothetical and requires targeted experimental validation.

Surface roughness was identified as an important factor in biofilm proliferation and the effectiveness of HIFU debridement in this study. The roughened titanium surfaces provided a more favourable environment for *S. mutans*, as evidenced by the CLSM z-stack images, which showed healthier and more abundantly embedded bacteria. Additionally, the amount of residual biofilm on these surfaces was significantly higher compared to machined surfaces. Jordana et al. [74] showed convincing evidence of the association between the level of implant roughness and the risk of peri-implantitis in their systematic review and meta-analysis.

The limitations of this study include its in vitro design, the small number of substrates, and the use of flat surfaces. In reality, dental implants present complex three-dimensional macrostructures and microscopic roughness that favour microbial colonization and complicate debridement. In oral settings, prostheses, anatomical constraints, and most importantly, peri-implant pockets, further hinder access for biofilm removal. As noted by Roccuzzo et al. (2024), the primary challenge of non-surgical peri-implantitis therapy is gaining adequate access to implant surfaces for effective decontamination [73]. Even on flat Ti surfaces, Tran et al. (2023), in their in vitro investigation, reported that none of the eight mechanical methods achieved complete biofilm removal, with the best result being a 96.53% reduction in MTT assay [23]. Consequently, the positive findings of this study should be interpreted with caution, and further research using PI-relevant multispecies biofilms in clinically representative models is necessary for validation and advancement. In our envisioned models, biofilms formed by Gram-negative anaerobic bacteria such as *F. nucleatum*, *P. gingivalis*, *P. intermedia*, and *T. forsythia* from the PI-associated “red complex” [75, 76] will be cultured and tested in three-dimensional peri-implant pocket models.

Additionally, improvements are needed in the dimensions and configuration of the HIFU transducer resembling a dental handpiece for intraoral experiments. In this experimental setting. Further optimization of HIFU parameters is essential to enhance the cavitation effect while minimizing hyperthermia. Regarding HIFU delivery to targeted biofilms, the manual operation of the transducer in our study introduces variables to the results and compromises the methodological reproducibility. Incorporating a guidance system into the HIFU transducer would enable the visualization and precise targeting of biofilms. In addition, mechanical fixation and standardized movements of the biofilms relative to the HIFU source are necessary. In future studies, we plan to use a guided HIFU transducer mounted at a fixed distance from the biofilm, which is moved in a programmed XY raster pattern via a mechanized platform.

In summary, the results from this study provide preliminary in vitro evidence that HIFU, given its potential advantages over existing non-surgical methods for debriding Ti implant-attached biofilms, merits further investigation as a possible therapeutic approach. These findings also support the continued use of dual fluorescence–reflection CLSM and FCM as valuable tools in dental implant biofilm research.

## Conclusion

Within the constraints of this in vitro mono-species biofilm model on flat Ti surfaces, HIFU effectively removed *S. mutans* biofilms. These results offer preliminary evidence that supports further methodological optimization and evaluation in clinically relevant settings, including multispecies biofilms and Ti implants within simulated peri-implant models.

## Data Availability

Supporting data have been provided in separate file alongside this submission.
